# Supplementary material for: Does Vaccination Protect against Human Papillomavirus-Related Cancers? Preliminary Findings from the United States National Health and Nutrition Examination Survey (2011–2018)
Source: Vaccines (Basel). 2022 Dec 10;10(12):2113. doi: 10.3390/vaccines10122113 (PMC9781459; doi:10.3390/vaccines10122113)
Supplement: Supplementary file 1 [file vaccines-10-02113-s001.zip › vaccines-2071274-supplementary.pdf]

Table S1. Descriptive characteristics of U.S. adults aged 20-59 stratified by HPV vaccination status using data from the National Health and Nutrition Examination Survey (NHANES), 2011-2018.

**Variables**	**Not HPV vaccinated (n=9,000)** **n (%)[a]**	**HPV vaccinated** **n (%)[a]**	**p-value[b]**
NHANES cycle			<0.001
2011-2012	2351 (26.0)	145 (14.7)	
2013-2014	2488 (25.7)	220 (22.4)	
2015-2016	2168 (24.2)	248 (27.2)	
2017-2018	1993 (24.2)	278 (35.8)	
HPV-related cancers, yes	134 (1.6)	6 (0.4)	<0.01
Age groups			<0.001
20-29 y.o.	1714 (18.5)	649 (76.8)	
30-39 y.o.	2351 (24.3)	152 (15.7)	
40-49 y.o.	2423 (27.4)	60 (4.9)	
50-59 y.o.	2512 (29.8)	30 (2.6)	
Sex, female	4259 (47.5)	687 (75.7)	<0.001
Education			<0.001
Incomplete high school	2447 (33.1)	271 (34.7)	
Complete high school	2013 (21.7)	158 (18.6)	
Some college or associate degree	1602 (12.5)	63 (5.5)	
College graduate or above	2938 (32.7)	399 (41.2)	
Ethnicity			0.30
Non-Hispanic White	2112 (15.6)	172 (14.0)	
Non-Hispanic Black	1978 (11.0)	211 (12.6)	
Hispanic	3515 (65.4)	348 (64.5)	
Other ethnicities	1395 (8.0)	160 (9.0)	
Born outside of US, yes	2431 (16.2)	133 (9.3)	<0.001
Not married, yes	3500 (34.8)	519 (56.7)	<0.001
Household income			0.06
<$25,000	3880 (39.6)	406 (44.6)	
$25,000-$75,000	2939 (43.7)	281 (38.1)	
>$75,000	2181 (16.7)	204 (17.3)	
Ever drank 4/5 alcohol drinks every day, yes	1472 (16.5)	82 (7.9)	<0.001
Smoked at least 100 cigarettes in life, yes	4121 (45.9)	274 (33.3)	<0.001
Self-reported diet, poor	3136 (31.3)	289 (28.8)	0.18
Ever been overweight, yes	4765 (54.4)	330 (35.3)	<0.001
History of diabetes, yes	925 (9.0)	32 (3.0)	<0.001

Moderate or vigorous physical activity at work, yes	4216 (49.4)	443 (53.8)	0.04
Routine access to healthcare services, yes	7032 (80.1)	729 (81.8)	0.36

Abbreviations: HPV, human papillomavirus; y.o., years old; US, the United States of America.

[a] Frequencies describe the study sample, while percentages represent survey weighted estimates for the US population.

[b] The Rao-Scott chi-square test for complex survey design was used to calculate p-values.

Table S2. Survey-weighted logistic regression models using complete-case, propensity score matched and multiply imputed datasets investigating the association of human papillomavirus vaccination history with HPV-related cancers (n=72) and other genital cancers (including prostate, testicular, cervical and ovarian cancers, n=68)[¶] among U.S. adults aged 20-59 in the National Health and Nutrition Examination Survey (NHANES), 2011-2018.

**Variables**	**aOR (95% CI)[a] Complete-case data[b]**	**Crude OR (95% CI) PS-matched data[c]**	**aOR (95% CI)[d] MI data[e]**
HPV vaccination history			
Not vaccinated	Reference	Reference	Reference
Vaccinated	0.50 (0.22; 1.13)	0.42 (0.15; 1.15)	0.41 (0.18; 0.95)*

Abbreviations: aOR, adjusted odds ratio; CI, confidence interval; OR, odds ratio, PS, propensity score; MI, multiple imputations; HPV, human papillomavirus.

¶ Participants who reported being diagnosed with oropharyngeal cancers such as larynx, windpipe, mouth, tongue, or lip; or anogenital cancers – cervical, prostate, testicular, cervical, or ovarian – were categorized as a HPV-related cancers and other genital cancers group while those who were not diagnosed with cancer or were diagnosed but with other cancer types were categorized as a no HPV-related cancer group.

[a] The survey-weighted multivariable logistic regression model was adjusted for age, sex, education, ethnicity, whether born in the U.S. or not, income, ever smoked at least 100 cigarettes, moderate or vigorous physical activity at work, history of diabetes, routine access to healthcare services and NHANES cycle. The goodness-of-fit of the model using the Archer-Lemenshow test p=0.05. The Area Under the Curve of the Receiver Operating Characteristics Curve was 0.80.

[b] The sample size in complete-case data was 9,891.

[c] Propensity score matched data included 861 cases diagnosed with head, neck or genital cancer and 861 controls who were not diagnosed with head, neck or genital cancer. Propensity score matching was performed using a 1:1 nearest-neighbor method (without replacement) with a caliper width of 0.2 of the standard deviation of the logit of the propensity score.

[d] The survey-weighted multivariable logistic regression model was adjusted for age, sex, education, ethnicity, marital status, whether born in the U.S. or not, income, ever smoked at least 100 cigarettes, moderate or vigorous physical activity at work, ever drank 4/5 alcohol drinks every day, history of diabetes, ever been overweight, routine access to healthcare services and NHANES cycle.

[e] Missing values for ever drank 4/5 alcohol drinks every day (25.7%), income (6.6%) and other covariates with less 1% missing data (routine access to healthcare services, self-reported diet, ever been overweight, marital status, smoked at least 100 cigarettes in life, education, moderate or vigorous physical activity at work, where born, history of diabetes) were imputed using “multiple imputation then deletion” approach. 20 imputations were used. Each imputed dataset contained 13,993 observations. Estimates were pooled using Rubin’s rules.

* p-value < 0.05

Table S3. Statistical power calculations based on the study sample size from the U.S. adults aged 20-59 in the National Health and Nutrition Examination Survey (NHANES), 2011-2018.

Cancer type	Number of not vaccinated	Prevalence among not vaccinated[a]	Number of vaccinated	Prevalence among vaccinated[b]	Statistical power[c]
Cervical cancer¶	4,259	0.003	687	0	30.1%
HPV-related cancers*	9,000	0.0063	900	0	66.1%

[a] Cancer prevalence estimates were calculated using data from the National Cancer Institute https://seer.cancer.gov/statfacts/

[b] We assumed that the vaccine would be highly effective (100%) against HPV-related cancers, so no cases would be detected in the HPV-vaccinated group.

[c] We calculated statistical power under two conditions: 1) when the outcome is only cervical cancer; 2) when the outcome is HPV-related cancers. In both conditions, we used two-sided alpha 0.05 to perform calculations. The statistical power estimates were calculated using the Epi Info online calculator available from https://www.openepi.com/SampleSize/SSCohort.htm

¶ Included only female participants

* HPV-related cancers including oropharyngeal (cancers of lip, oral cavity, pharynx, and larynx) and anogenital (cervical, vaginal, vulvar, penile) cancers.
